# An apparent motion color illusion

**DOI:** 10.1177/20416695241261147

**Published:** 2024-08-02

**Authors:** Rob van Lier, Simon J. Hazenberg, Vebjørn Ekroll

**Affiliations:** Donders Institute for Brain, Cognition and Behaviour, Radboud University, Nijmegen, The Netherlands; Department of Psychosocial Science, 87364 University of Bergen, Bergen, Norway

**Keywords:** color, apparent motion, visual illusion, filling-in

## Abstract

We introduce a new illusory color phenomenon. The illusion is evoked by two alternating displays comprising various colored disks. Although the colors in the alternating displays are the same, the color appearance of the two displays are quite different. We suggest that apparent motion of the disks modulates the color percepts.

Video 1.

In Video 1, the alternating displays are shown.^1^ When steadily fixating on the black dot for some time, the color appearance of the alternating displays left and right of the dot seems to differ for most observers. Typically, on the left, the colors appear mainly purple/pinkish, while on the right the colors appear mainly purple/blueish (see below for the results of a small experiment, revealing that for approximately 77% of the observers this is the case after at least 1 min of presentation with the instruction to fixate steadily). However, when observing the alternating displays in a more free-viewing fashion the displays look rather similar—and indeed, the colors of the disks left and right are exactly the same. What is different, however is the relative position of colored disks.

Let us take a closer look at how the displays are constructed (see Figure 1). The first frame in the movie is shown in A and the second frames are shown in B1 and B2. The purple disks in A can be regarded as a transparent superposition of the pink and the blue disks shown in B1 and B2. In B1 the pink disks remain at exactly the same position as the purple disks in A, while the blue disks are shifted sideways. In B2, it is the other way around: the blue disks remain at exactly the same position as the purple disks in A, while the pink disks are shifted sideways.

**Figure 1.**
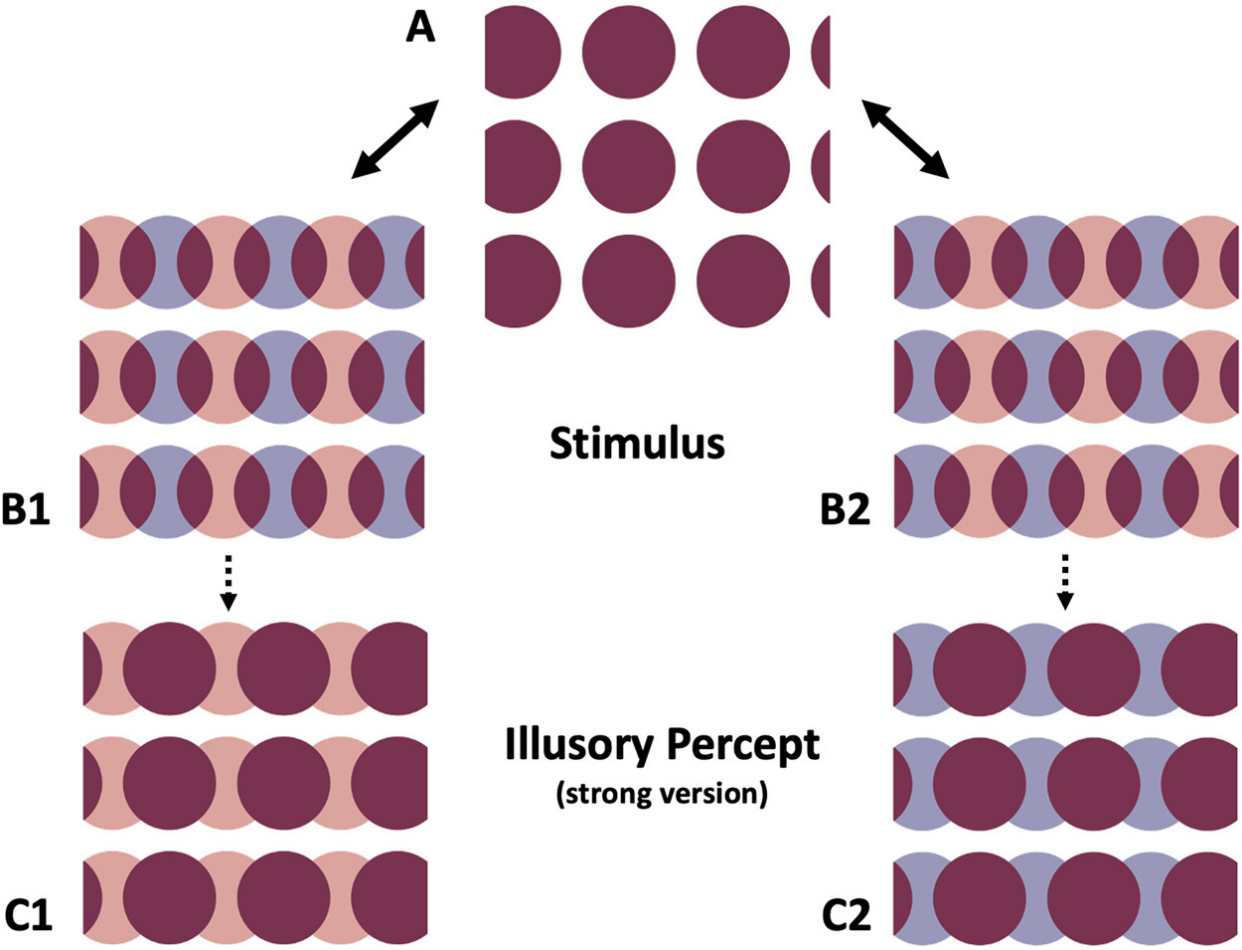
(A) Basic image comprising superimposed pink and blue disks. (B1) Splitting the colored disks by moving the blue disks sideways. (B2) Splitting the colored disks by moving the pink disks sideways. (C1, C2) Strong versions of the typical illusory percepts (the purple disks are often perceived to be moving, either back and forth or in one direction).

Back to Video 1: in the display on the left, A alternates with B1, while in the display on the right, A alternates with B2. Now, when steadily fixating on the black dot for some time, in both alternating displays the impression may arise that the purple disks move in a horizontal fashion. That is, during the alternation, there is a tendency to perceive purple disks in both color-split images, together with one of the other colors (pink or blue). See C1 and C2 for an indication of the illusory percepts. Please note that the actual percepts may not be as clear as in C1 and C2 as, e.g., often still a bit of blue can be seen in the A-B1 alteration and still some pink might be seen in the A-B2 alteration.

What is going on? We suggest that the illusory percept is the result of a color filling-in effect, triggered by the overlapping areas and the perceived motion of the purple disks (i.e., apparent motion, Anstis, 1980; Wertheimer, 1912). The filled-in purple disks cause one of the actually presented colors to mostly disappear, resulting in percepts akin to C1 and C2. In the A-B1 alteration, the filled-in purple disks coincide with the blue disks, causing the blue color to disappear, whereas in the A-B2 alteration, the filled-in purple disks cause the pink color to disappear.

We have initially shown the alternating displays at various scientific meetings such as the “illusion and demo night” at the ECVP 2022 in Nijmegen and quite a few people already confirmed the illusion as described here, but also with some individual differences in the percept and strength of the illusory percept. Therefore, we collected data by recruiting 30 students (22 females, 7 males, and 1 non-binary; age 17–33) of the Radboud University to participate in a brief experiment in our lab. Participants were randomly assigned to two conditions. In the original condition we used the sequence as shown in Video 1 (presenting B1 on the left of the fixation dot and B2 on the right of the fixation dot). In the reversed condition the position of the color-split images was reversed (presenting B1 on the right and B2 on the left of the fixation dot)^2^. After a short practice round, each participant was asked to fixate on the black dot while the movie was presented for 60 s. Afterwards, participants were asked what colors they saw using a forced choice with the answer options; “No color difference,” “On the left mainly blue/purple and on the right mainly pink/orange,” “On the left mainly pink/orange and on the right mainly blue/purple,” or “Color difference, but unsure which colors” (i.e., not as specified in the previous options). In addition, participants had the opportunity to replay the movie as often as needed in order to arrive at an answer.

The results are presented in Table 1. The majority of participants perceived a color difference and their answers strongly point in the direction of the illusory percept as described above. A few participants perceived the opposite color (seeing blueish where we expected pinkish and vice versa) and some perceived no color difference at all. When participants did notice a color difference, they did not have any difficulty describing the color difference. A chi-square test of independence yielded significant results, *χ*^2^ (2, *N *= 30) = 13.44, *p *= .001, implying that the answers depended on the condition.

**Table 1.** Number of Participants Choosing Answers Per Condition.

Condition	Frequency of participants			
	Left pink, right blue	Left blue, right pink	No color difference	Color difference, but unsure
Original	10	2	1	0
Reversed	2	13	2	0

Although the results are clear, they additionally show that the illusion was subject to individual differences, which also became apparent from remarks during a debriefing consisting of an explanation of the illusion and an additional viewing of the displays. For example, not all participants initially perceived apparent motion of the purple disks. Some became aware of apparent motion after the debriefing, but not all. Similarly, the strength of the color difference seemed to vary, presumably partly due to variability in the ability to steadily fixate and an uncertainty about what to look for. Indeed, some participants indicated a change in percept after the debriefing; one participant changed their answer from perceiving no color difference to perceiving the illusory color difference when emphasizing fixation and three participants changed their answer from perceiving a color difference in the opposite direction to a color difference in the suggested direction. The duration of the animation also appears crucial. During a pilot in which we showed the illusion for only 8 s, answers varied much more and results were less clear-cut, which indicates that it takes some time for the illusion to be fully established. Notice further that afterimages of the presented disks may also play a role. For instance, some participants reported that the perceived colors turned lighter or paler over time. This is likely caused by afterimages of the purple disks which are slightly darker than the pink and blue disks. In these cases, the effect does not seem to influence the perceived color difference, but it could be that for some participants the afterimages dominate the percept (perhaps due to differences in attention). All in all, these observations present interesting directions for further studies, as a variety of underlying mechanisms and differences in attention (e.g., focusing on apparent motion or focusing on afterimages) appear to drive the observed individual differences in how the illusion is perceived.

The phenomenon presented here adds to other phenomena that reveal an interaction between motion and color related processing (Cavanagh & Anstis, 1991; Chong et al. 2014; Cicerone & Hoffman, 2017; Hong & Kang, 2016; Gegenfurtner & Hawken, 1996). The motion-color interdependency clearly runs in both directions, from color to motion and vice versa. For example, Cavanagh and Anstis (1991) have shown that color contributes to motion perception by measuring color-dependent thresholds for the discrimination of moving colored gratings, while Chen and Cicerone (2002) describe the role of apparent motion to the formation of subjective colors (see also Cicerone & Hoffman, 2017). Although apparent motion appears to play a role in the current illusion as well, the displays are quite different. Perhaps the most noticeable perceptual difference is that the color impressions in the current illusion do not only relate to the apparently moving objects (i.e., the jumping “purple disks”), but also to the colors that are presented at the locations to which the purple disks appears to jump.

## Supplemental Material

**Figure:** Video 1.

## Notes

1. The video should best be viewed full screen. Take a viewing distance that is about double the size of the width of the movie clip.

2. Note that the quality of Figure 1 and the video have been enhanced for demonstration purposes, resulting in a slight difference in quality and colors compared to the video shown in the experiment (of which the RGB values were as follows; purple (126, 27, 68), blue (138, 130, 173), pink (227, 139, 134)).
